# Exercise in pregnant elite athletes: Physiological considerations, risks, and benefits

**DOI:** 10.14814/phy2.71148

**Published:** 2026-10-19

**Authors:** Ibelle Madera, Vedanti Bhargava, Jhillika Patel, Kritika Singh, Opal Jawale, Gloria Bachmann

**Affiliations:** ^1^ Johns Hopkins University Baltimore Maryland USA; ^2^ Women's Health Institute, Department of Obstetrics and Gynecology Robert Wood Johnson University Hospital New Brunswick New Jersey USA

**Keywords:** fetal health, maternal health, physical exercise, pregnancy, sports

## Abstract

The American College of Obstetricians and Gynecologists (ACOG) recommends engagement in moderate exercise throughout pregnancy, including aerobic and strengthening activities, which has been shown to offer numerous benefits during pregnancy. However, the intersection of pregnancy and athletics presents a unique set of challenges. ACOG guidelines on exercise during pregnancy primarily target recreational exercisers. Tailored recommendations are needed for pregnant elite athletes. This study examines both how pregnancy affects physiology and the risks/benefits of high‐intensity exercise. Two independent reviews were conducted, using MESH search terms including “pregnant athletes,” “female elite athlete,” “maternal health,” “exercise pregnancy.” From 56 total studies, 40 studies with clinical data on over 4163 women, including elite athletes, were included in the review. Regular exercise during pregnancy can improve cardiovascular fitness and fetal vascular function while reducing risks of gestational diabetes, hypertensive disorders, delivering a large‐for‐gestational‐age infant, and excessive weight gain. High‐intensity activities can compromise fetal blood flow. Endurance athletes may safely maintain their training regimens during pregnancy, whereas those in high‐altitude or contact sports require adjustments. Routine, non‐intense exercise during pregnancy offers significant maternal and fetal health benefits. Future work should establish individualized recommendations supporting pregnant elite athlete health.

## 1 INTRODUCTION

The American College of Obstetricians and Gynecologists (ACOG) recommends moderate exercise (e.g., aerobic and strengthening activities) throughout pregnancy, excepting medical contraindications. Non‐high‐intensity exercise offers numerous benefits during pregnancy, including improved cardiovascular fitness and reduced risk of gestational diabetes, hypertension, and excessive weight gain. However, the intersection of pregnancy and athletics presents a unique set of challenges.

As women's sports continue to advance, more women are pursuing athletic careers and may maintain high levels of physical activity during pregnancy. This raises important questions about how to support these women, who may engage in higher‐intensity or more prolonged activity than the average pregnant woman, through tailored guidelines that accommodate physiological changes during pregnancy while preserving athletic performance. Currently, most guidelines are generic, which limits their utility for professional athletes.

This review highlights the physiological relationship between exercise and pregnancy, with a particular focus on athletes.

## 2 METHODS

Two independent reviews were conducted in September 2025 via PubMed, using MESH search terms including “pregnant athletes,” “female elite athlete,” “maternal health,” “exercise pregnancy.” Studies were included if they contained primary data on athletes who were either pregnant or had recently delivered, with non‐pregnant women included as controls in some of the studies. Studies were excluded if they were case reports and included non‐human subjects. From 56 total studies, 40 studies with clinical data on over 4163 women, including elite athletes, were included in the review.

## 3 PHYSIOLOGY OF PREGNANCY AND EXERCISE

### 3.1 Changes in maternal physiology during pregnancy

Pregnancy induces changes across all maternal physiological systems. One notable alteration is increased metabolic demand, which contributes to maternal weight gain and elevated resting heart rate; however, maximal heart rate capacity decreases as gestation progresses (Ribeiro et al., 2021). Postural control is impacted, particularly after the first trimester, as the center of gravity shifts due to uterine expansion and breast hypertrophy, leading to altered gait biomechanics (Bø et al., 2017).

Hormonal fluctuations during pregnancy, including increased levels of progesterone, estrogen, and insulin, augment cardiac output and aortic compliance, ensuring adequate perfusion to both the mother and fetus. After approximately 20 weeks of gestation, the enlarging uterus can exert pressure on the aorta and inferior vena cava, leading to reduced venous return and potentially lowering cardiac output, a condition known as supine hypotensive syndrome (Bø et al., 2017; Lanni et al., 2002). These anatomical shifts may necessitate modifications in the type and intensity of physical activity that pregnant individuals can safely perform throughout pregnancy and at distinct points in pregnancy progression (Ribeiro et al., 2021).

### 3.2 Effects of exercise on maternal physiology during pregnancy

Engaging in exercise during pregnancy improves maternal cardiorespiratory capacity and fitness level, which may decrease maternal exhaustion, which typically increases throughout the course of pregnancy (Ribeiro et al., 2021). Pregnancy increases resting heart rate and decreases maximal heart rate, limiting the ability to adapt to exercise‐induced stress and leading to reduced heart rate reserve (Ribeiro et al., 2021). Physical activity that strengthens abdominal and back muscles could minimize the risk of experiencing low back pain, which is experienced by more than 60% of pregnant women due to an increase in forces across the spine and joints during weight‐bearing exercise (ACOG Committee Opinion, 2020).

### 3.3 Benefits and risks of exercise during pregnancy

Non‐high‐intensity exercise during pregnancy is safe and beneficial in preventing pregnancy‐related disorders. For instance, exercising during pregnancy can reduce the risk of acquiring several disorders unique to pregnancy on the mother and fetus, such as gestational diabetes mellitus (GDM), fetal macrosomia, weight retention in the postpartum period, hypertensive disorders, lumbopelvic pain, and urinary incontinence (Ribeiro et al., 2021). Lowering the likelihood of developing obesity and GDM subsequently reduces the risk of preterm birth. A combination of aerobic and resistance exercise improves maternal cardiorespiratory fitness (Perales et al., 2016). Additionally, exercise in general has been shown to reduce levels of anxiety and prenatal depression, benefiting overall maternal mental health (Ribeiro et al., 2021). Moderate, safe exercise does not increase the risk of adverse outcomes. No explicit risks of exercise during pregnancy are identified, and the risk of low birth weight and small gestational age is unrelated to exercise (Ribeiro et al., 2021). Moreover, greater health benefits have been observed with higher frequency and moderate intensity of exercise, which are often achieved in supervised exercise classes due to increased adherence to structured and guided exercise routines (Ribeiro et al., 2021).

### 3.4 Guidelines for exercising during pregnancy

ACOG recommends physical activity during pregnancy, with a combination of aerobic, strength, and resistance training, which improves cardiorespiratory fitness and reduces risk of urinary incontinence (ACOG Committee Opinion, 2020; Perales et al., 2016; Szumilewicz et al., 2022). Commonly recommended activities include walking, swimming, stationary cycling, dancing, and stretching (ACOG Committee Opinion, 2020). Pregnant women should avoid activities with high risks of abdominal trauma, falling, loss of balance, or those with physiological risk factors to protect both the mother and growing fetus (Bø et al., 2017).

Overall, exercise guidelines should be individualized to a woman's fitness level, medical history, and pregnancy characteristics (Perales et al., 2016; Ribeiro et al., 2021). Exercise contraindications include amniotic fluid leak, cervical insufficiency, multiple gestation, placental abruption, or placenta previa, premature labor, pre‐eclampsia/gestational hypertension, and severe cardiopulmonary disorders. Contact sports with a risk of abdominal trauma (e.g., ice hockey, basketball, soccer), high fall‐risk sports (e.g., downhill skiing, gymnastics, horseback riding), dehydration risk activities (e.g., hot yoga), and activities that decrease placental sufficiency (a common cause of preterm contractions) should be avoided. For uncomplicated pregnancies, 20–30 min of moderate‐intensity exercise is generally recommended to maintain overall fitness and well‐being (Figures 1 and 2).

**FIGURE 1:**
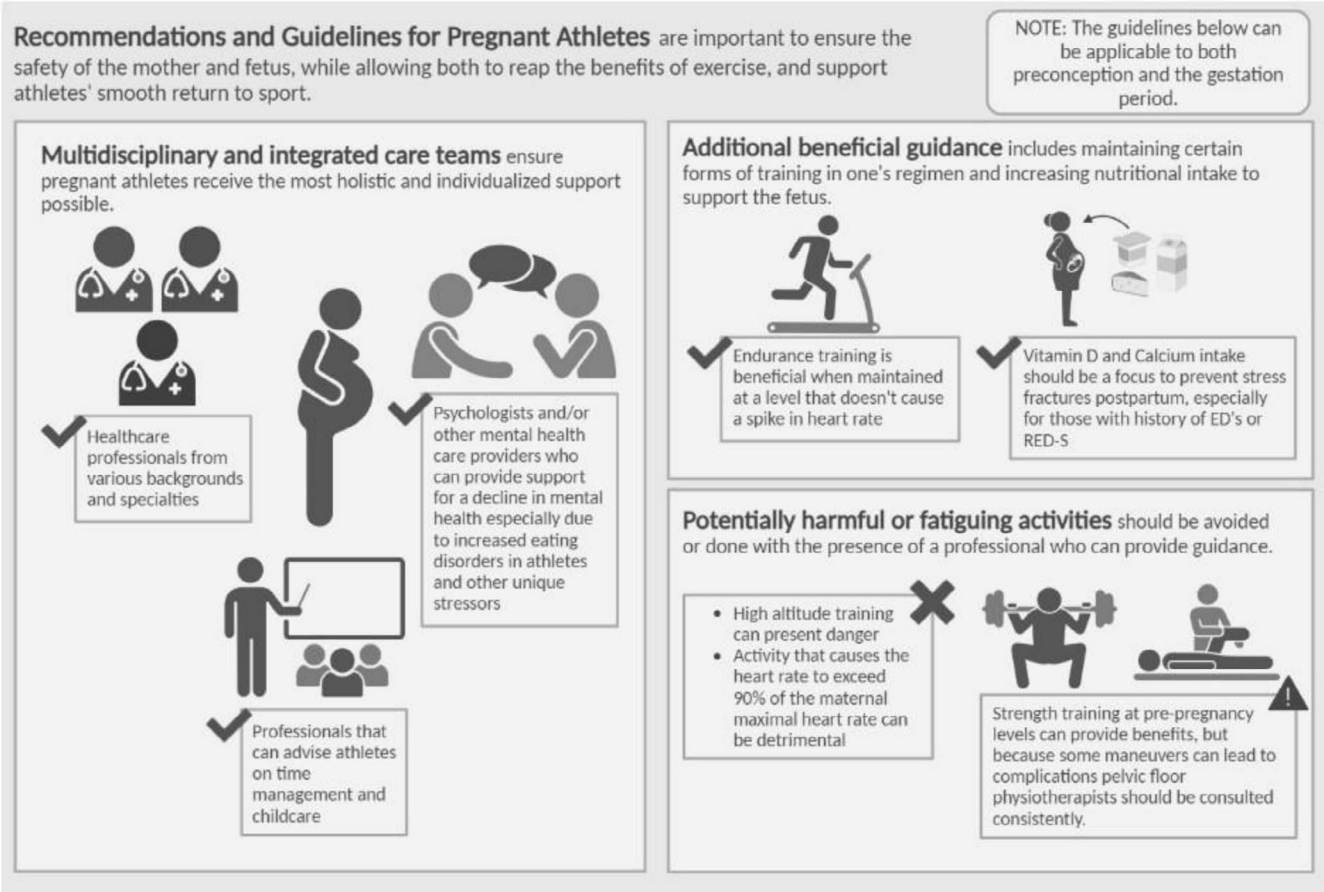
Recommendations and Guidelines for Pregnant Athletes. Figure created by Vedanti Bhargava and Jhillika Patel.

**FIGURE 2:**
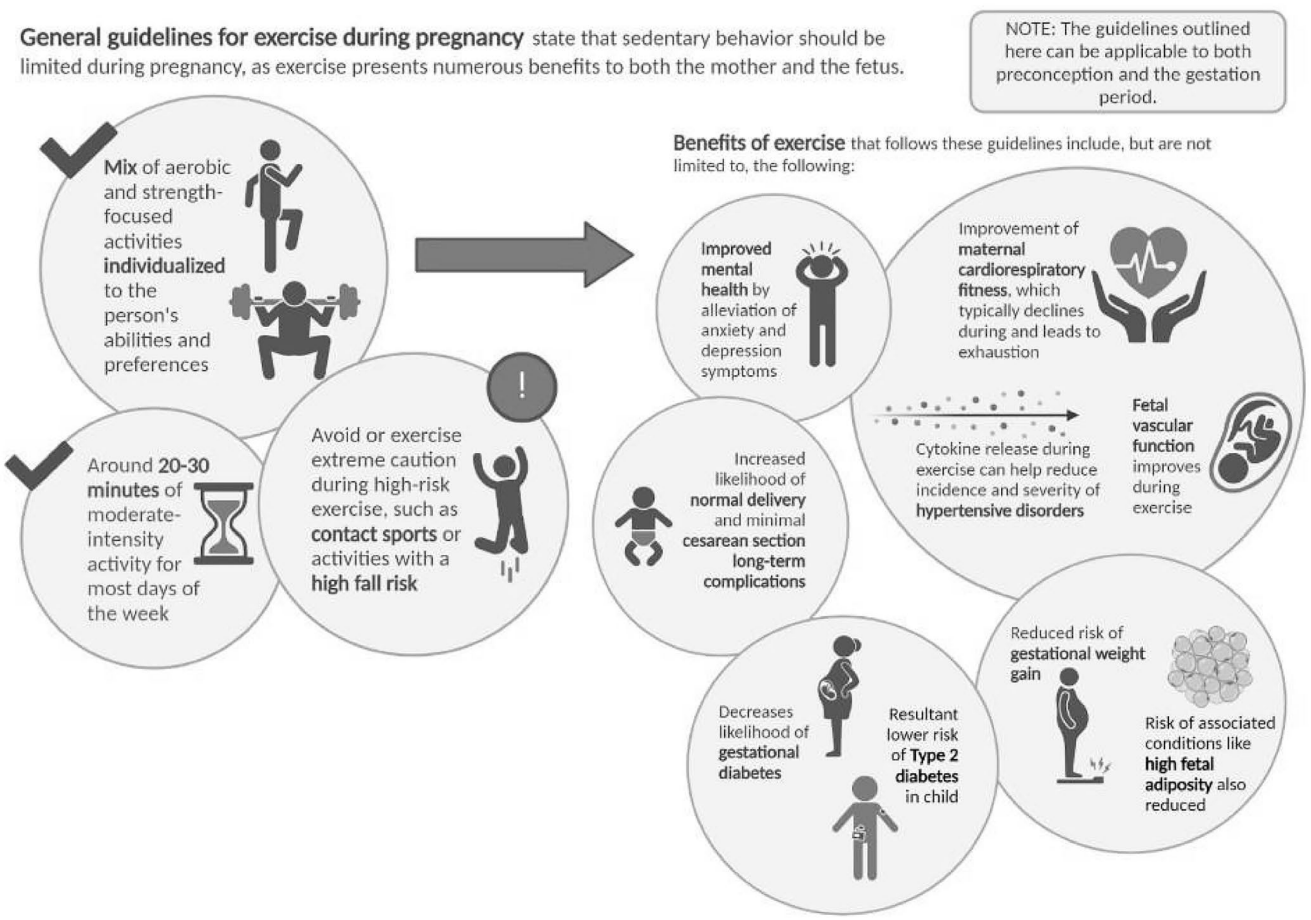
Summary of General Guidelines for Exercise during Pregnancy and Associated Benefits. Figure created by Vedanti Bhargava and Jhillika Patel.

## 4 IMPACT OF EXERCISE ON MATERNAL HEALTH OUTCOMES

Exercise interventions that last 12–24 weeks improve cardiorespiratory fitness, which otherwise declines throughout pregnancy, while reducing risks associated with sedentary behavior, including thromboembolism, bone demineralization, and negative psychosocial effects (ACOG Committee Opinion, 2020; Perales et al., 2016). Greater exercise volume and duration lower gestational weight gain, which often persists postpartum and raises the risk of cardiovascular disease and other chronic illnesses (Perales et al., 2016). Exercise lowers maternal and fetal leptin levels (which contribute to high fetal adiposity), which are significantly higher in early pregnancy among women who gain excessive weight (Vargas‐Terrones et al., 2019). Exercise reduces the risk of a large‐for‐gestational‐age infant from excessive pregnancy weight gain (da Silva et al., 2017; Vargas‐Terrones et al., 2019). Maternal exercise can positively impact fetal health by improving fetal vascular function, contributing to greater blood flow through the thoracic aorta; therefore, it improves endothelial function in the region and overall blood circulation (Blaize et al., 2015). While not comprehensively understood, regular exercise during pregnancy can modestly increase the likelihood of a normal delivery, avoiding health consequences associated with cesarean section deliveries (Poyatos‐León et al., 2015).

Non‐high‐intensity exercise lowers the risk of gestational diabetes, preeclampsia, and urinary incontinence, which can have long‐term adverse effects. While gestational diabetes is a normal complication during pregnancy, it can increase the risk of preeclampsia and cesarean section delivery, as well as the risk of developing type 2 diabetes within 10 years of pregnancy. Studies demonstrate the positive effects of all forms and intensities of exercise in reducing gestational diabetes (Paulsen et al., 2023). Preeclampsia is a less common (5%) but severe complication that can result in premature birth and long‐term cardiovascular disease for the mother; this condition can even threaten maternal and fetal life (Paulsen et al., 2023; Poniedziałek‐Czajkowska et al., 2023). Cytokines released during exercise have important metabolic and vascular effects, which can reduce the risk of hypertensive disorders during pregnancy (Poniedziałek‐Czajkowska et al., 2023). Pregnancy is a risk factor for stress urinary incontinence, a common condition among women that can substantially reduce maternal quality of life (Von Aarburg et al., 2021). This condition not only affects women during late gestation but can persist postpartum for up to one‐third of women with an uncomplicated pregnancy, and commonly occurs among patients with obesity as well (Ribeiro et al., 2021). The increased intra‐abdominal pressure and hormonal changes during pregnancy can injure pelvic floor muscles, leading to involuntary loss of urine during exertion, sneezing, or coughing. Aerobic exercises, as well as the integration of pelvic floor muscle training, can contribute to urinary incontinence prevention, especially by decreasing overall weight (Bø et al., 2017; Ribeiro et al., 2021). Overall, exercise can help prevent many serious complications of pregnancy, boost overall mental health, and improve cardiovascular fitness, serving as a key tool for a smoother postpartum recovery.

## 5 CONSIDERATIONS FOR ATHLETES

Athletes require individualized exercise regimens during pregnancy that support a return to sports postpartum without compromising fetal health. Given the overlap between peak athletic years and peak reproductive years, athletes face unique physiological, hormonal, and metabolic challenges (Santos‐Rocha & Szumilewicz, 2024). Energy intake must be increased accordingly with the higher energy expenditures of currently training pregnant athletes (Bø et al., 2017). Fatigue and nausea are commonly reported among pregnant women, resulting in dehydration, disrupted sleep, and impaired cognitive function. Most changes resolve after birth but can harm long‐term health, affecting the return to high‐intensity physical activity postpartum (Jackson et al., 2022). The maintenance of moderate‐level exercise during pregnancy can help reduce many of these long‐term maternal complications.

While moderate exercise is generally beneficial, high intensity exercise may compromise fetal health. Exercising at more than 90% maximal heart rate can lead to diminished fetal heart rate, high uterine artery pulsatility index, and a mean uterine artery volume blood flow less than 50% of the initial volume, all of which can result in fetal growth retardation (Salvesen et al., 2012; Wieloch et al., 2022).

Different forms of training require various considerations for pregnant athletes. Endurance training is among the most suitable for pregnant athletes (e.g., cross country) as moderate‐to‐high levels of exercise can maintain and even improve aerobic fitness. Athletes should avoid high‐altitude training (e.g., skiing, mountain biking) due to hypoxia risk, potentially compromising fetal oxygenation. Strength training (e.g., martial arts, bodybuilding) warrants caution; weight training could raise blood pressure and intra‐abdominal pressure which can impair fetal blood flow and increase the risk for urinary incontinence. Pregnant athletes should avoid sports with a risk of trauma (e.g., collision in hockey) or physiologic risk factors (e.g., scuba diving) (Bø et al., 2017).

There is a lack of definitive evidence of the impact of pregnancy on one's athletic performance. A study on 30 endurance athletes demonstrated that 11% achieved improved performance after pregnancy, 61% reached the same level, while 28% did not reach the same level (Penttinen & Erkkola, 1997). One common challenge is adapting to a “new body” postpartum while returning to sports. Breastfeeding, hormonal changes, and chronic fatigue can hinder the ability to return to previous athletic potential (Davenport et al., 2023). Prematurely returning to training postpartum can increase the risk of injury from elevated joint laxity during pregnancy and hormonal adaptations (high estrogen, relaxin, and progesterone) (Kimber et al., 2021). Postpartum bodily changes have led to greater rates of body dissatisfaction and drive for thinness among athletes compared to non‐athletes (Sundgot‐Borgen et al., 2019). These challenges and potential termination of an athletic career fuel high rates of anxiety, frustration, and depression among pregnant athletes, all of which are associated with poor birth outcomes (Bø et al., 2017).

## 6 PRACTICAL IMPLICATIONS AND RECOMMENDATIONS FOR PREGNANT AND POSTPARTUM ATHLETES

Preconception care for athletes requires consideration of the coincidence of peak fertility and peak athletic performance. Allen et al. outline a model for calculating the peak performance age of elite athletes to help athletes make better‐informed decisions. Competitive sports can create complications like relative energy deficiency in sport (RED‐S), which leads to impaired fertility. The higher prevalence of eating disorders (EDs) in athletes can lead to complications including preterm birth, spontaneous abortion, lower neonate Apgar scores, greater risk of vaginal bleeding, breech delivery, and more. As a result, multidisciplinary care teams are vital (Allen & Hopkins, 2015; Bø et al., 2017; Wieloch et al., 2022). These teams should include healthcare professionals and individuals who can help assemble postpartum training programs which include topics like time management and childcare advice (Beilock et al., 2001; Wieloch et al., 2022). Psychological support is vital due to unique mental health considerations for athletes and because athletes with higher motivation and stronger efficacy beliefs in training during pregnancy tend to return to competition sooner (Beilock et al., 2001; Ribeiro et al., 2021).

Some data demonstrate the benefit of intensive strength training in alleviating pregnancy‐associated complications and conditions that disproportionately affect athletes (Beilock et al., 2001). Perinatal depression and incidences of cesarean section seemed to be lower in those who did heavy weightlifting during pregnancy in one study. Those who maintained pre‐pregnancy strength training levels compared to those who lowered training intensity or completely stopped showed a lower incidence of reproductive and delivery complications (Bø & Backe‐Hansen, 2007; Sigurdardottir et al., 2019). Support for intensive strength training, including the Valsalva maneuver, supine weightlifting, and Olympic weightlifting while pregnant, is preliminary (Prevett et al., 2023). For high levels of strength training, it may be beneficial to obtain guidance and pelvic floor muscle training from pelvic floor physiotherapists to safely maintain training intensity during pregnancy, prevent urinary incontinence, lower rates of prolonged labor, and reduce the number of women with breech presentation and episiotomies (Sundgot‐Borgen et al., 2019).

Although it is difficult to generalize guidelines on an ideal regimen and training intensity level from existing data, interdisciplinary teams should work to ensure that appropriate activity levels are maintained to allow a smooth transition back into sport (Allen & Hopkins, 2015; Wieloch et al., 2022). Training efforts during pregnancy may be independent of pre‐ and post‐partum regimens, indicating that training patterns can be altered without a significant detriment to postpartum training (Beilock et al., 2001).

### 6.1 Guidelines for exercising safely during pregnancy for athletes

Due to their more intense activity levels, athletes must take certain additional precautions to exercise safely during pregnancy. Because their energy expenditure is likely higher, athletes' nutritional requirements must be matched to prevent deficits (Bø et al., 2017). During the postpartum period, there seems to be an increase in stress fractures, possibly due to a sudden increase in training load. Greater focus should be placed on increasing bone strength through adequate energy intake and preventative increased consumption of calcium and vitamin D, particularly for athletes with a history of RED‐S or an ED (Sundgot‐Borgen et al., 2019).

While limited research around strength training exists in athletes specifically, it has been demonstrated that the Valsalva maneuver during intense weight training can decrease blood flow to the fetus (Prevett et al., 2023). This is largely based on expert opinion as opposed to empirical evidence, as there are recent studies demonstrating that such high‐intensity strength training is well‐tolerated by the mother and fetus (Gould et al., 2021; Moolyk et al., 2025; Prevett et al., 2023). However, increased intra‐abdominal pressure during heavy strength training can lead to urinary or anal incontinence or pelvic organ prolapse—though it is expected in non‐pregnant women as well (Wieloch et al., 2022). Athletes engaging in such exercise (especially strenuous strength training) show they have similar rates of low back pain, pelvic girdle pain, and urinary incontinence during pregnancy as non‐athletes (Bø & Backe‐Hansen, 2007; Sundgot‐Borgen et al., 2019). In more recent studies, light to moderate strength training shows no adverse effects and rather leads to strength gains (Beilock et al., 2001).

Training intensity also requires consideration. Athletes training at high altitudes must be cautious, as lower oxygen levels combined with exercise could lead to a detrimental lack of blood flow to the fetus (Bø et al., 2017). Intensity above 90% of the maternal maximal heart rate could be detrimental to the fetus, although further research is needed (Salvesen et al., 2012; Santos‐Rocha & Szumilewicz, 2024; Sundgot‐Borgen et al., 2019). There is research in support of elite athletes with uncomplicated pregnancies maintaining their training intensity to maintain fitness levels postpartum. Kardel et al. outline a training regimen that facilitated a relatively easy return to sport for athletes postpartum, but the results need to be replicated with a larger population (Kardel, 2005). Endurance training has not been shown to have harmful side effects on pregnancy or delivery in athletes (Penttinen & Erkkola, 1997). Retrospective studies have also shown that the training levels of elite athletes are not necessarily associated with more complicated pregnancies including higher rates of emergency cesarean section, prolonged second stage of labor, and third‐degree to fourth‐degree perineal tears (Wowdzia et al., 2021).

### 6.2 Future research directions in the field

There is a lack of studies that assess factors unique to athletes during preconception, pregnancy, and postpartum; many research directions can be pursued specifically in support of pregnant athletes.

Limited data exists on conditions unique to or more prevalent in athletes in the preconception stage, such as RED‐S and eating disorders (Bø et al., 2017). The long‐term implications of RED‐S are unknown. Current studies lack representative samples on EDs in pregnant athletes (Bø et al., 2017). While some data indicate no significant difference in fertility, average infant birth weights, miscarriages, and preterm births for athletic versus non‐athletic mothers, more studies are required to corroborate (Davenport et al., 2022; Sundgot‐Borgen et al., 2019). Additional areas of research that need to be addressed include the impact of training on the menstrual cycle, as it is closely related to fertility (Carmichael et al., 2021; L'Heveder et al., 2026; Taim et al., 2023).

Physiological changes and associated precautions remain largely unexplored in athletes. The effect of exercising in the supine position (which may cause hypotension in the general pregnant population), metabolic changes, strength and flexibility training changes, high altitude exercise, overhead Olympic weightlifting, repeated use of the Valsalva maneuver, and endurance changes in pregnant athletes are all lacking in studies (Bø et al., 2017; Mottola et al., 2019; Redondo‐Delgado et al., 2025). The safe threshold of strength training (when compared to the percent maximum aerobic capacity) is yet to be determined. Regarding strength training specifically, research concerning blood pressure changes related to heavy loads is needed. Both athlete and non‐athlete pregnant people report dissatisfaction with the current scope of guidance provided for strength and intense endurance training (Sundgot‐Borgen et al., 2019; Wieloch et al., 2022).

Many of the clinical complications that come with pregnancy are understudied in athletes, including nausea prevalence (which can be associated with EDs), depression and anxiety, gestational weight gain, the effect of exercise in reducing fatigue, gestational hypertension, edema, musculoskeletal complaints (e.g., pelvic girdle pain and low back pain), and intervention and prevention of pelvic floor dysfunction (especially its relation to strength training). Studies on biomechanical changes in pregnancy in both regular exercisers and elite athletes remain few (Bø et al., 2017). Research on long‐term neurological consequences of children repeatedly exposed to high‐intensity exercise as fetuses is limited (Wieloch et al., 2022). A While it has been established to some degree that maternal exercise during pregnancy can reduce the risk of certain chronic diseases in offspring, studies need to have more diversified exercise intensity (Blaize et al., 2015). The high prevalence of low back pain, pelvic girdle pain, and urinary incontinence in both athlete and non‐athlete pregnancies, highlights the need for the implementation of actionable strategies (Bø & Backe‐Hansen, 2007; Kardel, 2005).

Another major sector of research to address includes establishing a timeline for the appropriate return of physical activity for postpartum athletes. This involves ensuring that athletes optimize their time during their years of peak athletic performance but do not compromise fetal and maternal health. Studies must assess if an earlier return is associated with increased risk of injury postpartum or changes in athletic performance compared to the pre‐pregnancy stage (Kimber et al., 2021). Furthermore, the 90% heart rate intensity threshold, which has demonstrated potential detriment to the fetus due to acute fetal bradycardia (Santos‐Rocha & Szumilewicz, 2024; Sundgot‐Borgen et al., 2019) and elevated pulsatility index of the umbilical artery, needs to be further explored to clarify clinical significance (Mone et al., 2016; Salvesen et al., 2012).

There is a great need to establish evidence‐based recommendations regarding sports activity for elite athletes prioritizing their sport. More randomized control trials and observational studies that focus on the female athlete population, with sufficient power and sample size, are necessary to fill this gap in the literature (Kimber et al., 2021; Wieloch et al., 2022). It follows that any results from future studies should be translated into guidelines that are used to educate coaches, healthcare providers, and athletes (Wieloch et al., 2022).

### 6.3 Policy implications and advocacy for supporting pregnant athletes

There is a lack of knowledge among professionals who work with athletes (e.g., sports medicine doctors, coaches) regarding the physiological and biomechanical processes unique to pregnant athletes may prevent them from receiving proper guidance around maintaining physical activity, from competing during and after pregnancy, and could potentially cause a delayed return to their career (Santos‐Rocha & Szumilewicz, 2024). This could entail the withdrawal of sponsorships, which may be particularly stressful, as paid leaves of absence during or after pregnancy are uncommon for elite female athletes (Darroch et al., 2019; Kimber et al., 2021; Santos‐Rocha & Szumilewicz, 2024). In qualitative studies, athletes' express hesitation in disclosing their pregnancy to coaches and sports organizations for fear of being discredited in their careers (Davenport et al., 2022). Studies also show a high rate of athletes ending their elite careers to focus on their children (Zahl Marken et al., 2025).

Sports organizations need to adjust policy so that pregnant athletes are supported and can attain the same level of career success as athletes who do not get pregnant. Studies show specific areas where policy change could be beneficial: allowing more time for return‐to‐play following childbirth (especially when considering injuries that may result from labor), accounting for physical, mental, and emotional postpartum changes in developing training regimens, supporting breastfeeding, and providing financial support. Any financial policies and maternity leave policies that organizations have should be made clear to athletes, so they can make informed decisions on when or whether to get pregnant. There is a need to recruit more professionals who can provide support in each of these areas, such as lactation consultants or pelvic floor physiotherapists. Development of anti‐discrimination policies seems vital, especially in removing the fear athletes feel around disclosure or intent of pregnancy (Davenport et al., 2023; Jackson et al., 2022).

Focusing on the knowledge gaps around the impact of exercise at elite athletic level on pregnancy, it is important to return to the overall gender gap in data and research; advocacy efforts should be directed at increasing the representation of women in scientific literature (Jackson et al., 2022). Amplifying the voices of pregnant elite athletes is a key step in boosting confidence and driving away cultural perceptions, which often paint athletes in a negative light for endangering the fetus if they participate in sport. Efforts to build networks of athletes considering, in, and past pregnancy can help with this goal, as social support is vital to greater athletic engagement during this time period (Davenport et al., 2022; Martinez‐Pascual et al., 2014; Zahl Marken et al., 2025).

## 7 STRENGTHS AND LIMITATIONS OF EXISTING RESEARCH

Though studies examining and surveying human participants have examined a wide range of factors, fewer than a thousand elite‐level athletes were surveyed. While the relationship between exercise and the cardiovascular and metabolic changes inherent to pregnancy is well understood, the mechanisms that underlie the impact of exercise on maternal weight gain and the likelihood of normal delivery have not been fully defined. Understanding the correlation between exercise and health outcomes is useful for providing accurate, personalized healthcare to elite athletes competing in a variety of sports.

## 8 CONCLUSIONS

Current guidelines for women during pregnancy encourage moderate exercise to maintain health and fitness amidst the array of physiological changes that occur during pregnancy. While overall increased frequency and intensity of exercise are associated with greater health benefits, exercise regimens need to be individualized, especially if there is planned ongoing athletics participation.

Tailored approaches, including input from sports medicine and obstetrics professionals, are vital to ensure that athletic women can continue to train safely and effectively throughout their pregnancy. Interdisciplinary care models and ongoing research into pregnancy and exercise are necessary.

Given that the age of peak athletic performance and peak fertility overlap, elite athletes may become pregnant while competing. Often the upkeep of their fitness is tantamount to career maintenance, but the potential effects of strenuous exercise need to be taken into account to ensure health. Just as recommending “moderate” exercise sets different expectations for an average woman and an elite athlete, female athletes in different sports engage in wildly varied forms of training and must adapt to pregnancy accordingly. Some sports may be ideal for exercise during pregnancy, while others may carry risks. Pregnant athletes must meet additional nutritional requirements to compensate for increased energy costs and increase bone strength to maintain the standard of health needed to continue exercising throughout pregnancy and return to the sport safely postpartum. Maintaining physical health is often linked to an athlete's mental health due to higher rates of body dissatisfaction and concerns about returning to athletic careers in the case of non‐maintenance of physical health. Therefore, elite athletes must maintain fitness during pregnancy not only for their physical health but also for the optimal development of the fetus and the preservation of their mental health. Though this review provides insight into the relationship between maternal health and exercise, further research is needed, especially when considering both the health and careers of female athletes. Studies are needed across all stages of pregnancy from preconception to postpartum. With further data, athletes and sports organizations can develop personalized training regimens to maintain maternal physical and mental health, optimal fetal development, and peak athletic performance.

## AUTHOR CONTRIBUTIONS

**Ibelle Madera:** Data curation; formal analysis; investigation; methodology. **Vedanti Bhargava:** Data curation; formal analysis; methodology. **Jhillika Patel:** Conceptualization; data curation; formal analysis; investigation; methodology; project administration; resources; supervision. **Kritika Singh:** Data curation; formal analysis; investigation; methodology. **Opal Jawale:** Data curation; formal analysis; project administration. **Gloria Bachmann:** Conceptualization; data curation; formal analysis; methodology; supervision.

## FUNDING INFORMATION

The authors have nothing to report.

## CONFLICT OF INTEREST STATEMENT

The authors declare no conflicts of interest.

## ETHICS STATEMENT

The authors have nothing to report.

## CONSENT

The authors have nothing to report.

## Data Availability

The datasets generated during and/or analyzed during the current study are available from the corresponding author on reasonable request.
